# 1984. Effects of Semaglutide on Adipose Tissue in HIV-Associated Lipohypertrophy

**DOI:** 10.1093/ofid/ofad500.111

**Published:** 2023-11-27

**Authors:** Grace A McComsey, Abdus Sattar, Zainab Albar, Kianoush Ansari Gilani, Danielle Labbato, Theresa Foster, Allison Ross Eckard

**Affiliations:** Case Western Reserve University , Cleveland, OH; Case Western Reserve University, Cleveland, OH; Case western reserve university school of medicine, cleveland, Ohio; University Hospitals Cleveland Medical Center, Cleveland, Ohio; University Hospitals, Cleveland, Ohio; University Hospital, Cleveland, Ohio; Medical University of South Carolina, Charleston, South Carolina

## Abstract

**Background:**

Lipohypertrophy (central adipose tissue (AT) accumulation) is a common and significant problem in people with HIV (PWH). Pathogenesis remains elusive; yet, AT abnormalities are key drivers of cardiometabolic co-morbidities in HIV. We aimed to assess effects of semaglutide, a glucagon-like peptide-1 receptor agonist, on AT in PWH with lipohypertrophy.

**Methods:**

We conducted a randomized, double-blinded, placebo-controlled trial of virologically-suppressed, non-diabetic PWH ≥ 18 years of age on stable antiretroviral therapy (ART) with body mass index (BMI) ≥ 25 kg/m^2^, increased waist circumference/waist-to-hip ratio, and subjective increased abdominal girth after ART initiation. Participants were randomized 1:1 to 32 weeks semaglutide (8-week titration + 24 weeks 1.0 mg weekly subcutaneous injection) or matching placebo. Computed tomography and whole-body dual-energy X-ray absorptiometry were used to measure area/density in abdominal AT [total (TAT), visceral (TAT), and subcutaneous (SAT)] and body composition [lean body mass (LBM), limb/trunk/total body fat (TBF)], resp. Semaglutide effects were estimated using generalized estimating equations or simultaneous quantile regressions on outcome variables.

**Results:**

108 participants were enrolled (N = 54 semaglutide: median age = 52 years, 70% male, 61% Black, 83% integrase inhibitor). Groups were well-matched at baseline. In unadjusted models, semaglutide group had greater reductions (P < 0.05) in BMI, homeostatic model of insulin resistance (HOMA-IR), trunk fat, TBF (at quantile ≥ 75^th^), TAT, and SAT with trends (P < 0.1) for limb fat and VAT (Fig 1/Table 1). Semaglutide effects remained significant for BMI, HOMA-IR, trunk fat, TAT, and VAT after adjusting for age, sex, CD4, and ART duration (Table 2); caloric intake was also significant at ≤ 50^th^ quantile. No differences were seen in LBM, AT density, or VAT/TAT ratio. Semaglutide was well-tolerated; serious adverse events were rare.

**Figure:**
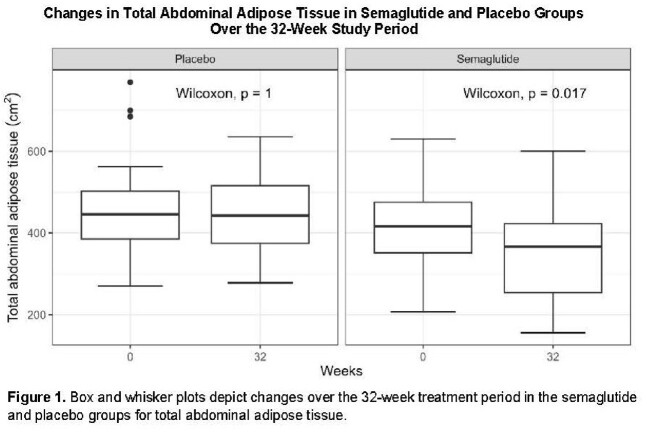

**Figure:**
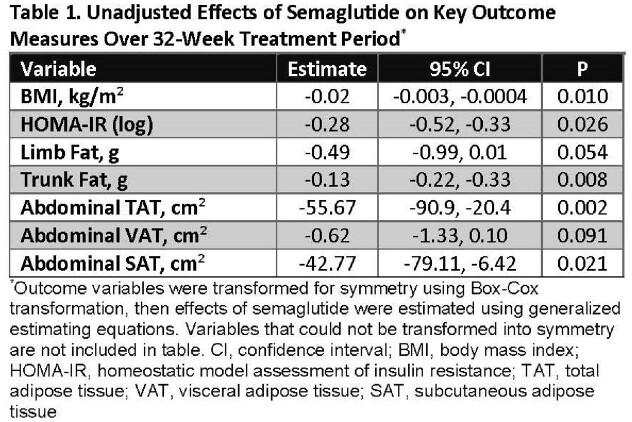

**Conclusion:**

Semaglutide significantly decreased central fat in PWH with lipohypertrophy, primarily driven by reductions in VAT. Semaglutide may offer an effective treatment to decrease visceral adiposity and reduce co-morbidity risk. Further investigation is needed to determine mechanisms by which reductions in visceral adiposity occur.

**Figure:**
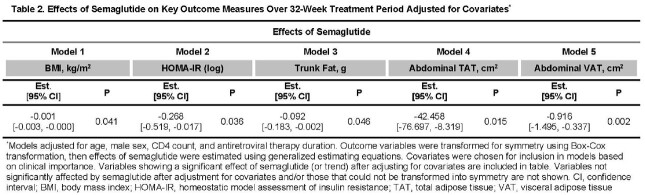

**Disclosures:**

**Grace A. McComsey, MD**, Gilead Sciences: Advisor/Consultant|Gilead Sciences: Grant/Research Support|Janssen: Advisor/Consultant|Merck: Advisor/Consultant|Merck: Grant/Research Support|ViiV Healthcare: Advisor/Consultant|ViiV Healthcare: Grant/Research Support

